# The Roles of Coinhibitory Receptors in Pathogenesis of Human Retroviral Infections

**DOI:** 10.3389/fimmu.2018.02755

**Published:** 2018-11-27

**Authors:** Keiko Yasuma-Mitobe, Masao Matsuoka

**Affiliations:** ^1^Department of Microbiology and Immunology, Keio University School of Medicine, Tokyo, Japan; ^2^Department of Hematology, Rheumatology and Infectious Disease, Faculty of Life Sciences, Kumamoto University, Kumamoto, Japan; ^3^Laboratory of Virus Control, Institute for Frontier Life and Medical Sciences, Kyoto University, Kyoto, Japan

**Keywords:** co-inhibitory receptor, HTLV-1, HIV-1, PD-1, TIGIT

## Abstract

Costimulatory and coinhibitory receptors play a key role in regulating immune responses to infection and cancer. Coinhibitory receptors include programmed cell death 1 (PD-1), cytotoxic T-lymphocyte-associated protein 4 (CTLA-4), and T cell immunoglobulin and ITIM domain (TIGIT), which suppress immune responses. Coinhibitory receptors are highly expressed on exhausted virus-specific T cells, indicating that viruses evade host immune responses through enhanced expression of these molecules. Human retroviruses, human immunodeficiency virus (HIV) and human T-cell leukemia virus type 1 (HTLV-1), infect T cells, macrophages and dendritic cells. Therefore, one needs to consider the effects of coinhibitory receptors on both uninfected effector T cells and infected target cells. Coinhibitory receptors are implicated not only in the suppression of immune responses to viruses by inhibition of effector T cells, but also in the persistence of infected cells *in vivo*. Here we review recent studies on coinhibitory receptors and their roles in retroviral infections such as HIV and HTLV-1.

## Introduction

Various viruses cause acute and chronic infections in humans. Since the host immune system functions to eliminate exogenous virus and infected cells, viruses that cause chronic infections must evade host immune surveillance by various strategies. Some viruses that cause persistent infections are also associated with viral oncogenesis; these include hepatitis C virus (HCV) (1), hepatitis B virus (HBV) (2, 3), human T-cell leukemia virus type 1 (HTLV-1) (4, 5), and Epstein-Barr virus (EBV) (6). Human immunodeficiency virus (HIV) also contributes to the development of cancers (7).

Costimulatory and coinhibitory receptor molecules play a key role in regulating immune responses to infections and cancers (8). When bound by their ligands, coinhibitory receptors suppress excess immune responses. In several cancers, tumor-infiltrating T cells express coinhibitory molecules that enable the tumors to escape the host immune response. Recent studies show that antibodies that block coinhibitory receptors, called immune checkpoint blocking antibodies, enhance immune responses to various cancers and exhibit remarkable clinical efficacy in cancer treatment (9).

Both virus-specific T cells and tumor-infiltrating T cells express coinhibitory molecules, and immune checkpoint pathways play a role in maintaining an exhausted T cell phenotype characterized by impaired cytokine production and cytotoxicity (10). The attenuated responses cannot eliminate viruses. In this review, we focus on coinhibitory receptors in retroviral infections.

## Coinhibitory receptors

An increasing number of coinhibitory molecules and pathways have now been identified (Table 1). There are two major families of T cell cosignaling molecules: the immunoglobulin superfamily (IgSF), which includes the B7-CD28 subfamily, and the tumor necrosis factor superfamilies of ligands (TNFSF) and receptors (TNFRSF). The B7-CD28 subfamily includes the classic coinhibitory receptor cytotoxic T-lymphocyte-associated antigen 4 (CTLA-4) (35). B7 family molecules, such as CD80 and CD86, enhance TCR-mediated responses through binding to the co-stimulatory receptor CD28. On the other hand, CTLA-4 competes with CD28 for binding to CD80 and CD86 (36), thus playing a critical role in regulating T-cell activation and expansion (37–39). Thus, coinhibitory CD28 subfamily molecules negatively regulate T cell responses (8). Another representative molecule of this subfamily is programmed cell death 1 (PD-1: also known as Pdcd1). The IgSF also includes T cell immunoglobulin and mucin domain 3 (Tim-3), T cell immunoglobulin and ITIM domain (TIGIT), Lymphocyte activation gene-3 (Lag-3), and 2B4 (CD244, a member of the signaling lymphocyte activation molecule (SLAM) family of CD2-related receptors) (32, 40–42).

**Table 1:** Inhibitory Ig superfamily and TNF superfamily receptors and their stimulatory molecules expressing during retrovirus infection^*^.

**Supperfamily**	**Receptor subfamily**	**Molecules**	Expression in infection^**^	**Receptor expressing cells during infection**	**Signaling**	**Known ligands**	**References**
Ig SF	CD28	CTLA-4	HIV	CD4+	Inhibitory	CD80, CD86	11, 12
		PD-1	HIV, SIV, HTLV-1	CD4+, CD8+	Inhibitory	PD-L1, PD-L2	12–18
		BTLA	HTLV-1 (decreased)	CD4+ (ATL cells)	Inhibitory	HVEM, UL144	19
	CD226	TIGIT	HIV, SIV, HTLV-1	CD4+, CD8+	Inhibitory	CD155, CD112, CD113	18, 20
	TIM	Tim-3	HIV, HTLV-1 (decreased)	CD4+, CD8+	Inhibitory	Galectin9, PS	21–26
	CD2/SLAM	2B4(CD244)	HIV, HTLV-1	CD8+	Stimulatory/inhibitory	CD48	27–29
	LAIR	LAIR1	HTLV-1 (decreased)	CD4+ (ATL cells)	Inhibitory	Collagens	19
	Orphans	Lag-3(CD223)	HIV	CD4+	Stimulatory/inhibitory	MHC2/ unknown	30
		CD160	HIV	CD8+	Stimulatory/inhibitory	HVEM	27, 28
TNFRSF	Type-L	HVEM	HIV	Monocytes, DCs	Stimulatory	LIGHT	31
					Inhibitory	BTLA, CD160

* *This table is based on modified 32–34*.

** *Decreased expression is mentioned specifically, otherwise expression is elevated during indicated infection*.

The other major group of cosignaling molecules, members of the TNFSF and TNFRSF, elicit costimulatory and coinhibitory signals between various cells. Most TNFRSF members bind to their specific TNFSF ligands and elicit costimulatory signals; however, herpesvirus entry mediator (HVEM) binds to several different ligands, including both TNFSF members and IgSF members. These ligands provide both stimulatory and inhibitory signals from HVEM. Binding of HVEM to the IgSF member B and T lymphocyte attenuator (BTLA) triggers inhibitory signals (43). HVEM also binds to the IgSF member CD160 and elicits a coinhibitory signal (44). CD160 is involved in both NK cell activation and T cell exhaustion (43). Another TNFRSF member, Death receptor 6, has also been reported to be a regulatory receptor (45). Coinhibitory receptors that are expressed during retrovirus infection are shown in Table 1.

## T cell exhaustion and viral infection

When T cells are chronically activated by viral infections, T cells tend to express coinhibitory receptors, and acquire exhausted phenotypes. Although most murine retroviruses establish chronic infection only when they infect neonatal mice, Friend virus (FV) causes acute and chronic infection even when it infects adult immunocompetent mice, suggesting that FV can evade the host immune responses and cause persistent infection (46). Regarding this, FV is similar to HIV and HTLV. As well as lymphocytic choriomeningitis virus (LCMV) infection (47, 48), FV-specific effector CD8 T cells express multiple coinhibitory receptors, such as PD-1, Tim-3, Lag-3, and CTLA-4 during chronic FV infection. Those cells were shown to be dysfunctional and associated with exhaustion (49, 50). In murine retrovirus model of FV chronic infection, blocking of CTLA-4 showed augmented T cell response and decreased the viral load (51). In addition to enhanced expression of coinhibitory receptors, regulatory T (Treg) cells also increase, which is associated with inhibition of effector T cells during FV infection (49, 52, 53). Thus, combined treatment of depletion of Treg cells and blockade of coinhibitory receptors recover CD8 T cell responses to FV (52).

Although FV infection is one model of retrovirus infection, it should be pointed out that FV is a retrovirus which infects mainly erythroid precursor cells and causes erythroleukemia. The target cell type is different from that of human retroviruses, such as HIV and HTLV-1, which target immune cells including T cells. Next, we review the coinhibitory receptors and their roles in pathogenesis of human retrovirus infections, HIV and HTLV-1.

## Retrovirus infection and coinhibitory receptors

Coinhibitory receptors are also implicated in persistent infection with human retroviruses, HIV, and HTLV-1. However, one difference between these viruses and most others is that the target cells of these human retroviruses are the immune cells themselves, including T cells, macrophages and dendritic cells—cells that also express coinhibitory receptors. Moreover, inhibitory ligands are also expressed on retrovirus infected cells (54), which can cause dysfunction of effector cells through interaction with coinhibitory receptors. Therefore, we need to consider the effects of coinhibitory receptors on two types of cells: uninfected effector cells to the virus, and cells that are infected with the virus.

## Human immunodeficiency virus (HIV)

To established chronic infection, human retroviruses have to evade the host immune response. One mechanism is the escape mutations of epitopes that are recognized by cytotoxic T lymphocytes (CTL). Since viral reverse transcriptase is an error-prone DNA polymerase, vigorous viral replication generates vast number of mutations in the provirus. If the target epitope of CTL is mutated, this mutation enables the virus to escape from CTL responses. Furthermore, HIV impairs the immune function of effector cells to HIV infected cells through co-inhibitory receptors, which also helps virus to escape from immune responses (55). We are going to discuss about several co-inhibitory receptors.

### PD-1

**Figure 1:**
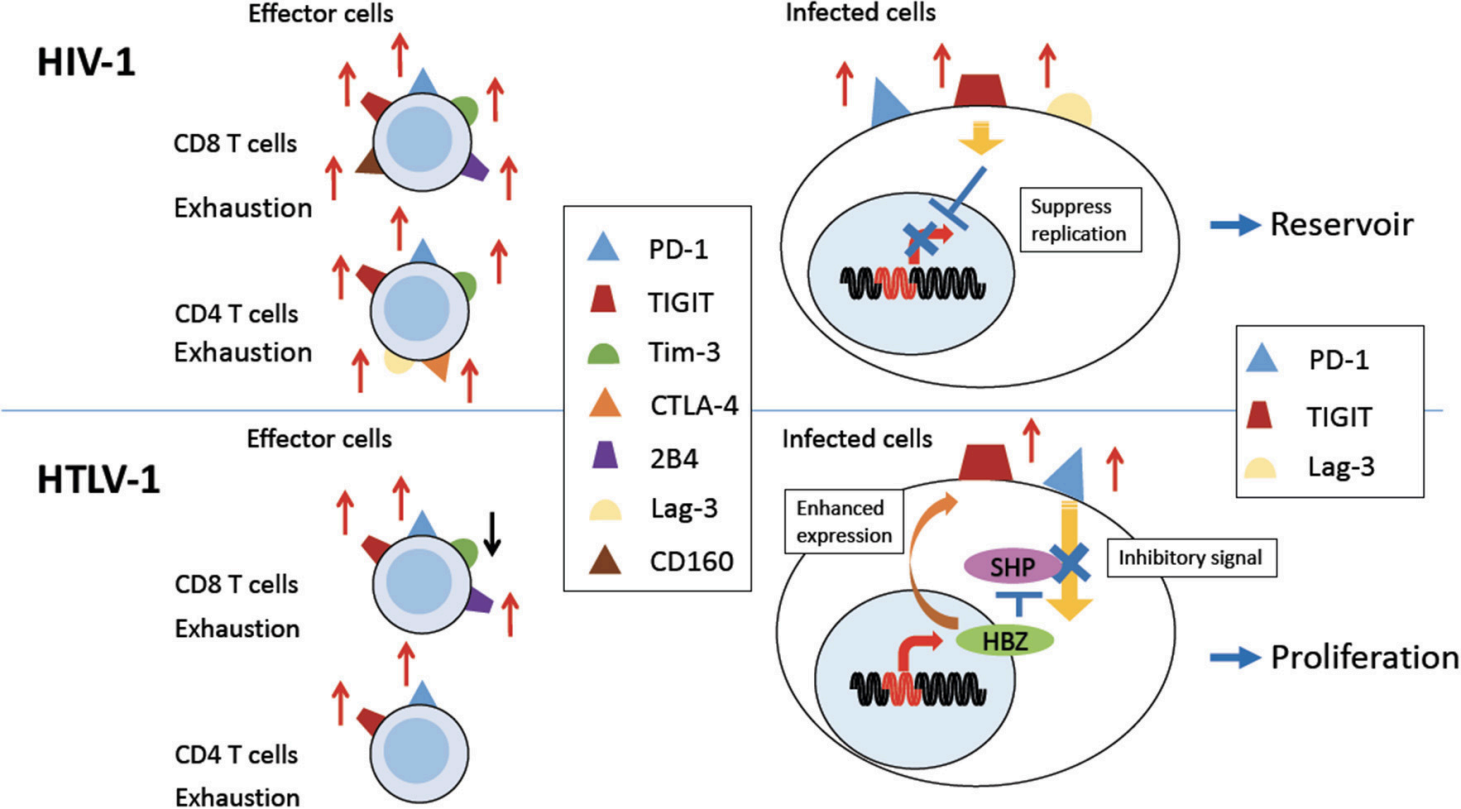
Expression of coinhibitory receptors in HIV-1 and HTLV-1 infection. Persistent HIV-1 **(Upper Left)** and HTLV-1 **(Bottom Left)** infection induces expression of various coinhibitory receptors on uninfected effector CD8 T cells, and some uninfected CD4 T cells, causing exhaustion of T cells (left). PD-1 and TIGIT and/or Lag-3 are also expressed on HIV-1 or HTLV-1 infected CD4 T cells (right). In HIV-1 infection, coinhibitory receptor expression is implicated in establishment of a viral reservoir **(Upper Right)**. In HTLV-1 infection, expression of coinhibitory receptors is enhanced by the viral protein HBZ. Inhibitory signals from coinhibitory receptors are impaired by HBZ. Thus, infected cells are able to proliferate despite of increased expression of coinhibitory receptors **(Bottom Right)**.

PD-1 expression is upregulated on HIV-specific CD8 and CD4 T cells in humans (Figure 1, upper left). The expression of PD-1 on these cells is positively correlated with viral load and disease progression (13), suggesting that PD-1 expression allows viral replication *in vivo*. HIV infection also upregulates PD-L1 expression on infected cells, which impairs T-cell function through interaction with increased PD-1 on effector T cells (54). Blockade of the PD-1/PD-L1 pathway using antibodies against PD-L1 *ex vivo* restores the function of HIV-specific CD4 and CD8 T-cells from anti-retroviral therapy naïve patients (13). Further studies investigated the effect of blocking the PD-1 pathway using an *in vivo* mouse model. The effect of PD-L1 blocking antibodies was analyzed in humanized mice chronically infected with HIV-1. The blockade of the PD-1 pathway decreased HIV-1 viral loads and suppressed disease progression, especially in animals with high levels of PD-1 expression on CD8 T cells (14, 15). A recent study showed that antibodies targeting BTLA and Tim-3 in combination with PD-1 antibody also enhanced HIV-specific CD8 T cells proliferation *in vitro* (56). These studies suggest that the blocking of these coinhibitory receptors is an effective strategy to restore the anti-virus T cell responses and suppress viral load in HIV-infected individuals. In particular, this strategy combined with “shock-and-kill” therapy and/or ART might be beneficial for control of HIV.

The SIV infected rhesus macaque is the *in vivo* model of HIV-1 infection. An *in vivo* experiment using rhesus macaques also showed that PD-1 blockade enhances SIV-specific CD8 T cell responses, reduced viremia, and prolonged survival of SIV-infected macaques (57, 58), especially in combination with antiretroviral therapy (ART) (31).

### CTLA-4

CTLA-4, another inhibitory receptor, is also upregulated in HIV-specific CD4 T cells, most of which co-express it with PD-1 (11) (Figure 1, upper left). CTLA-4 expression also positively correlates with disease progression. Blocking of CTLA-4 enhances HIV-specific CD4 T cell proliferation in response to HIV protein (11).

### Tim-3

The exhaustion of HIV-specific CD8 T cells is also mediated by Tim-3 (Figure 1). The frequency of Tim-3 expressing dysfunctional T cells was elevated in HIV-1 infected individuals. In particular, Tim-3 expression was upregulated in HIV-specific CD8 T cells. Tim-3 expression was positively correlated with viral load and inversely correlated with CD4 T cell count (21). Tim-3 triggers cell death after interaction with its ligand, Galectin-9 (Gal-9) (22–24). Treg cells constitutively express Gal-9 and suppress proliferation of HIV-specific CD8 T cells with high level of Tim-3 expression (59). Furthermore, Tim-3 expressing HIV-specific CD8 T cells are defective in regard of degranulation (25). It has also been reported that PD-1, CTLA-4, and Tim-3 are co-expressed on HIV-specific CD4 T cells from untreated infected patients, and the co-expression of these three inhibitory receptors was strongly correlated with viral load (12).

### TIGIT

TIGIT is often coexpressed with PD-1 at higher levels on HIV-specific CD8 T cells in HIV-infected patients, and this expression correlates with exhaustion of T cells and disease progression (Figure 1). TIGIT is highly expressed on intermediately differentiated memory CD8 T cells that are not fully mature effectors, which expand in HIV infection (20, 60). It has been reported that TIGIT+ cells produce less IL-2, TNF-α and IFN-γ and degranulate less (20). In addition, TIGIT expression on CD4 T cells is also associated with HIV viral load. As was the case for the other inhibitory receptors described above, blocking TIGIT and/or PD-L1 restores CD8 T cell responses *in vitro* (20).

### Other inhibitory receptors in HIV infection

Other inhibitory molecules are also implicated in HIV infection. HIV-specific CD8 T cells expressing PD-1 also express CD160 and 2B4 (27, 28) (Figure 1). Co-expression of these inhibitory receptors correlates with virus load and T cell responses. ART reduces the expression of these inhibitory molecules on the surfaces of HIV-specific CD8 T cells. Furthermore, blocking of the PD-1/PD-L1 and 2B4/CD48 pathways enhances the proliferation of virus-specific CD8 T cells (27). Higher expression of Lag-3 on CD4 T cells was also reported in rapid progressors of HIV infection (30) (Figure 1, upper left).

HVEM, which is a member of the TNFRSF and the ligand of CD160, is upregulated on monocytes and dendritic cells (DCs) in HIV chronically infected patients. Blocking the interaction between CD160 and HVEM also enhanced the proliferation of HIV-specific CD8 T cells (61).

### Implication of coinhibitory receptors for persistence of HIV

So far we have discussed the effects of coinhibitory receptors on uninfected effector T cells to HIV (Figure 1, upper left). Next we need to consider the effects of these coinhibitory receptors on HIV infected cells themselves. Coinhibitory receptors suppress the proliferation and activation of infected cells (62) (Figure 1, upper right). First, PD-1 has been shown to associate with persistence of HIV in patients under ART treatment (63). The expression of the co-inhibitory receptors PD-1, TIGIT, and LAG-3, correlates with HIV-infected cells persisting in individuals treated with ART (64) (Figure 1, upper right). Thus, these studies suggest that co-inhibitory receptors play a important role in the formation of an HIV reservoir. It has been clarified that more than 95% of HIV-1 provirus in patients who receive ART are defective (65, 66). Such defective provirus does not kill infected cells since it cannot produce infectious virion. However, infected cells with defective provirus can produce viral proteins, which cause inflammation. It is likely that continuous inflammation upregulates PD-1, resulting in immune exhaustion and escape of infectious HIV-1 from host immune responses.

## Human T-cell leukemia virus type 1 (HTLV-1)

HTLV-1 causes adult T-cell leukemia (ATL) and inflammatory diseases including HTLV-1-asscociated myelopathy/tropical spastic paraparesis (HAM/TSP) (4, 67). The *HTLV-1 bZIP factor* (*HBZ*) gene plays a critical role in oncogenesis and inflammation. HBZ is constantly expressed in ATL cells and HTLV-1-infected cells in carriers, and furthermore, transgenic expression of HBZ induces T-cell lymphomas and systemic inflammatory diseases resembling those found in HTLV-1-infected individuals (68, 69). One prominent feature of HTLV-1 is that this virus transmits primarily through cell-to-cell contact. To facilitate its transmission, HTLV-1 increases the number of infected cells *in vivo*. Therefore, this virus has evolved strategies to promote the proliferation of infected cells and evade host immune surveillance. Although HTLV-1 can infect a variety of cells, it primarily infects CD4^+^CD45RO^+^CCR4^+^ T cells *in vivo*. It is thought that HTLV-1 increases this special subtype of CD4^+^ T cells *in vivo*. HBZ is thought to be critical for this special phenotype since HBZ converts infected cells to this special phenotype T cells (70).

### Coinhibitory receptors in HTLV-1 infection

Since HTLV-1 mainly infects CD4 T cells *in vivo*, we again need to consider the roles of coinhibitory receptors on two different kinds of cells: infected CD4 T cells and uninfected effector T cells (Figure 1). It has been reported that during chronic HTLV-1 infection, PD-1 expression is increased on HTLV-1-specific CD8 T cells (Figure 1) (16). At the same time, ATL cells and HTLV-1 infected CD4 T cells of HAM/TSP patients express high levels of PD-1 (17, 18). These infected cells also express high levels of TIGIT (Figure 1) (18). Interestingly, the expression of PD-1 and TIGIT is enhanced in HBZ gene-transduced T cells (18), whereas the expressions of other coinhibitory receptors, BTLA and LAIR-1, is suppressed (19). This selective enhanced expression of particular coinhibitory receptors appears to be unique to HTLV-1. Co-blocking of PD-1 and TIGIT *ex vivo* partially restores anti-Tax T-cell responses in some HAM/TSP patients.

Flow cytometry showed that PD-L1 expression is also upregulated in 21.7% of ATL cases (16). One possible mechanism of the upregulation of PD-L1 has been identified: 27% of ATL cases possess structural variations that commonly disrupt the 3' untranslated region of the *PD-L1* gene, resulting in increased *PD-L1* transcripts (71). This upregulated PD-L1 expression enables ATL cells to evade the host CTL responses by causing exhaustion of effector T cells.

### Immune impairment by coinhibitory receptors on HTLV-1 infected cells

HBZ-induced coinhibitory receptors on HTLV-1 infected cells likely impair anti-virus T cell responses (18). As a mechanism, high expression levels of TIGIT promote production of IL-10 from CD155 positive DCs by reverse signaling (i.e., signaling from coinhibitory receptor ligands, such as PD-L1 and/or PD-L2 for PD-1, and CD155 for TIGIT on DCs). IL-10 not only suppresses the host immune response, but also promotes proliferation of HTLV-1 infected cells and ATL cells (72). The reverse signaling reduces maturation of DCs and changes them to a suppressive phenotype (73, 74). Furthermore, TIGIT competes for the binding of CD155 with CD226, a stimulatory receptor on T cells (75). Thus, HTLV-1 induces expression of coinhibitory receptors on effector T cells not only by chronic infection resulting in exhaustion of effector T cells but also by direct effect of HBZ on HTLV-1 infected T cells (18).

### HBZ-mediated escape from the growth suppressive effect of coinhibitory receptors

Coinhibitory receptors normally inhibit the proliferation of expressing T cells through binding of their ligands (76). However, the enigma is that ATL cells and HTLV-1 infected T cells do proliferate *in vivo*. Coinhibitory receptors like PD-1 and TIGIT normally inhibit cell proliferation through the ITIM or ITSM domains of their cytoplasmic regions, which interact with the phosphatases SHP1 and SHP2. However, HBZ expressing cells are resistant to the growth-inhibitory effects of TIGIT. THEMIS forms complexes with Grb2 and SHP and recruits them to the ITIM or ITSM domains of the coinhibitory receptors (77). HBZ interacts with THEMIS and impairs the growth-suppressive signal through SHP (Figure 1, bottom right). Thus, HBZ induces the expression of coinhibitory receptors while it blocks their suppressive effects on the proliferation of expressing cells (19).

### IL-10

Mice with impaired IL-10 signaling were reported to develop autoimmune colitis, suggesting a critical role of IL-10 in regulating inflammation (78). IL-10 has various effects on many hematopoietic cells: IL-10 causes dendritic cells to downregulate stimulatory IL-12 production and expression of costimulatory molecules (78, 79). In addition, IL-10 decreases T-cell cytokine production (78). Thus, IL-10 is critical for suppressing immune responses. High levels of TIGIT induce IL-10 production, leading to a suppressed host immune response (74).

### Anti-PD-1 antibody for treatment of ATL

Recently, it has been reported that anti-PD-1 antibody induced rapid progression of ATL after its administration (80). It has been reported that PD-1 functions as a tumor suppressor in T-cell lymphomas (81). This is the case in some ATL cases. This finding suggests that HBZ partially hinders the suppressive effects of PD-1. Thus, the significance of coinhibitory receptors for ATL cells needs further study.

### CD244 and tim-3

Another inhibitory receptor, 2B4 (CD244), is expressed at elevated levels on CD8 T cells in HTLV-1 infected patients and especially on HTLV-1 specific CD8 T cells. Blockade of the interaction between 2B4 and its ligand CD48 by antibody to CD48 *in vitro* enhances HTLV-1 specific CD8 T cell effector function measured by increased CD107a degranulation and perforin expression (29). On the other hand, Tim-3 expression is reduced on CD4 and CD8 T cells of HTLV-1 infected patients (26). This selective expression may be modulated by virus genes, such as HBZ, in order to have an advantage of virus survival.

## Perspectives and conclusion

Since exhausted T cells are also implicated in chronic viral infections as described in this review, immune checkpoint therapy could be a novel treatment for diseases associated with persistent viral infections as well as anti-tumor therapy (82). Notably for human retroviral infections, coinhibitory receptors on both effector cells and infected target cells play different roles in the pathogenesis. Coinhibitory receptors on target cells infected with HIV or HTLV-1 likely promote their survival by protecting target cells from immune responses or inhibiting viral production. Further studies are necessary to clarify the roles of coinhibitory receptors in chronic viral infections.

## Author contributions

All authors listed have made a substantial, direct and intellectual contribution to the work, and approved it for publication.

### Conflict of interest statement

The authors declare that the research was conducted in the absence of any commercial or financial relationships that could be construed as a potential conflict of interest.
